# Temporal Trends and Geographic Variability in the Prescription of Antiretroviral Treatments in People Living with HIV in Spain, 2004–2020

**DOI:** 10.3390/jcm11071896

**Published:** 2022-03-29

**Authors:** Marta Ruiz-Algueró, Victoria Hernando, María Riero, José Ramón Blanco Ramos, Miguel Alberto de Zarraga Fernández, Pepa Galindo, Alexandre Pérez-González, Asunción Díaz, Inés Suárez-García, Inma Jarrín, CoRIS cohort

**Affiliations:** 1 National Center for Epidemiology, Institute of Health Carlos III, 28029 Madrid, Spain; vhernando@isciii.es (V.H.); adiaz@isciii.es (A.D.); ijarrin@isciii.es (I.J.); 2 CIBER de Enfermedades Infecciosas, Instituto de Salud Carlos III, 28029 Madrid, Spain; 3 Complejo Hospitalario de Navarra, 31008 Pamplona, Spain; maria.rivero.marcotegui@navarra.es; 4 Hospital San Pedro Centro de Investigación Biomédica de la Rioja (CIBIR), 26006 Logroño, Spain; jrblanco@riojasalud.es; 5 Hospital Universitario de San Agustín, 33401 Avilés, Spain; migueldezarraga@gmail.com; 6 Hospital Clínico Universitario de Valencia, 46010 Valencia, Spain; galindo.pepa1@gmail.com; 7 Hospital Álvaro Cunqueiro, 36213 Vigo, Spain; alexandre.perez@iisgaliciasur.es; 8 Infectious Diseases Group, Department of Internal Medicine, Hospital Universitario Infanta Sofía, FIIB HUIS HHEN, 28703 Madrid, Spain; inessuarezgarcia@gmail.com; 9 Universidad Europea de Madrid, 28670 Madrid, Spain

**Keywords:** antiretroviral therapy, trends, HIV infection, cohort studies

## Abstract

Background: The purpose of this study was to describe temporal trends in the use of antiretroviral therapy (ART) among people living with HIV (PLWHIV) from the cohort of the Spanish HIV/AIDS research network (CoRIS), 2004–2020. Methods: We described the yearly evolution of the proportion of patients receiving ART and the most frequently prescribed antiretroviral drugs among newly recruited treatment-naïve patients and among all patients with active follow-up. Results: Of 15,539 patients included, 14,618 (94.1%) started ART during their follow-up. Regarding initial regimens, the use of 2NRTI plus 1NNRTI (which were the most frequently prescribed until 2014) and 2NRTI plus 1bPI decreased after 2014, being gradually replaced by INI-based triple therapies. Since 2019, other regimens started to be prescribed, mainly dual therapies. TDF/FTC/EFV was the single-tablet regimen (STR) most frequently prescribed as initial ART until 2012, decreasing thereafter as TDF/FTC/RPV, TDF/FTC/EVG/COBI, and ABC/3TC/DTG became available. TAF/FTC/BIC accounted for 53.6% of initial prescriptions in 2020, followed by DTG/3TC (24%). The percentage of patients on ART increased from 45.7% in 2004 to 98.2% in 2020. Among all patients receiving ART, regimens based on 2NRTI plus 1INI increased from 0.1% in 2007 to 53.3% in 2020. During 2007–2015, most patients were receiving TDF/FTC/EFV, which was replaced after 2017 by ABC/3TC/DTG. In 2020, 13.0% of patients were receiving dual therapies. Conclusions: Robust real-world data on ART use in PLWHIV over more than 15 years show historical trends in prescriptions with an unprecedented visualization of the contemporary treatment patterns.

## 1. Introduction

According to the latest UNAIDS estimations, 37.7 million people globally were living with HIV in 2020 and 27.5 million people were receiving antiretroviral therapy (ART) [1]. Today, all persons living with HIV are dependent on life-saving ART that suppresses HIV replication, prevents the development of AIDS, and reduces the risk of transmission [2].

Thirty-five years after the approval of the first HIV treatment, AZT (Zidovudine), multiple ART regimens are recommended for treatment-naïve and -experienced HIV-positive individuals based on efficacy and tolerability data obtained from randomized controlled trials and cohort studies [3]. Several of the older treatments are no longer used in clinical practice, since they have been displaced by new, more potent, less toxic drugs with a more convenient dosage regimen. Presently, a wide range of therapeutic options provides clinicians with extensive choices while the clinical research agenda for ART is now shifting towards addressing new questions: long-acting formulations, transdermal drug delivery systems, novel therapeutic targets, treatment regimens seeking to minimize long-term toxicity such as two-drug ART regimens, and ways to ensure the sustainability of long-term ART (such as providing affordable and effective treatments with generic antiretroviral drugs), among others [4,5].

In Spain, health care is decentralized to its 17 autonomous regions and two autonomous cities. Each region establishes its own health plans and has the responsibility for the budget setting, organization, delivery, and evaluation of health services within its territory. ART is prescribed by hospital clinicians and provided free of charge in the public health care system to all patients in the hospital pharmacies. In previous studies, we have shown that the choice of ART can be influenced by a variety of factors, not only dependent on the patient but also on the prescribing physician, budget limitations, and hospital characteristics [6,7,8]. In this regard, ART purchases and acquisitions by the different public hospitals in Spain follow different procedures. Since 2015, many autonomous regions have chosen to centralize their antiretroviral purchases in an attempt to homogenize their prices in every hospital and thus reduce expenditures. However, adherence and application of this centralized purchase are uneven and coexist with other complex drug procurement procedures [9,10].

To our knowledge, there are no real-world published data in Spain on the use of ART and prescribed regimens over time, or the geographic variations in ART prescriptions at the country level. We sought to describe temporal trends and geographic variability in the use of ART and the prescription of regimens to individuals with HIV infection from a large Spanish multicenter cohort, in the period 2004–2020.

## 2. Materials and Methods

### 2.1. Study Population

We included antiretroviral-naïve individuals recruited in the cohort of the Spanish HIV/AIDS research network (CoRIS) cohort from 1 January 2004 to 30 November 2020. CoRIS is an open, prospective, multicenter cohort of subjects with confirmed HIV infection, naïve to ART at study entry, recruited in 47 centers from 14 of the 17 autonomous regions in Spain from 2004 onwards. Briefly, CoRIS collects a minimum dataset which includes baseline and follow-up socio-demographic, immunological, and clinical data including ART [11]. Data are highly standardized and submitted to periodic quality control procedures. Patients are followed up with periodically in accordance with routine clinical practice.

The administrative censoring date for these analyses was 30 November 2020. For this study, two centers were excluded because they were neither recruiting new patients nor providing follow-up data for previously recruited ones, and one center was excluded because no ARTs were prescribed in it.

### 2.2. Statistical Analysis

Descriptive analysis of patients’ characteristics was carried out using frequency tables for categorical variables and medians and interquartile ranges for continuous variables. Differences in socio-demographic and clinical characteristics at ART initiation according to the period of starting ART (2004–2007, 2008–2011, 2012–2015, and 2016–2020) were assessed with the non-parametric Kruskal–Wallis test for continuous variables and the chi-squared test for independence for categorical variables.

We calculated the yearly evolution for the period 1 January 2004 to 30 November 2020 in (i) the number and percentage of patients initiating ART, overall and according to the year of enrollment in the CoRIS cohort; (ii) the prescription of first-line antiretroviral regimens, individually and by type of regimen in this group; (iii) the percentage of patients receiving ART among those in active follow-up; and (iv) the ART regimens received, individually and by type of regimen in this group. ART regimens were classified as two nucleoside/nucleotide reverse transcriptase inhibitors (NRTIs) plus one non-nucleoside reverse transcriptase inhibitor (NNRTI), two NRTIs plus one boosted protease inhibitor (bPI), two NRTIs plus one integrase strand transfer inhibitor (INI), dual therapies, other combinations, and unknown (i.e., regimens received in the context of double blind clinical trials).

When patients had received more than one regimen in the same year, only the first regimen was considered. To assess the geographic variability in the use of ART, we calculated these indicators in each of the 14 autonomous regions. For the sake of simplicity, analyses on geographic variability were restricted to the last three years (2018, 2019, and 2020).

All statistical analyses were performed using Stata software (version 15.0; Stata Corporation, College Station, TX, USA).

## 3. Results

During the study period, 15,539 patients were recruited in CoRIS, among which 14,618 (94.1%) started ART during their follow-up; this percentage varied by autonomous region, ranging from 90.7% to 100.0%.

Patients’ characteristics at ART initiation changed over time (Table S1). The percentage of women decreased from 24.3% in 2004–2007 to 12.2% in 2016–2020. Median age decreased from 38.1 in 2004–2007 to 35.8 in 2016–2020, and the percentage of people who injected drugs decreased from 18.2% to 2.4% for the same periods. The percentage of patients born in Spain decreased from 60.7% to 55.2%, while the proportion of those born in Latin America increased from 15.7% to 27.9%.

**Figure 1:**
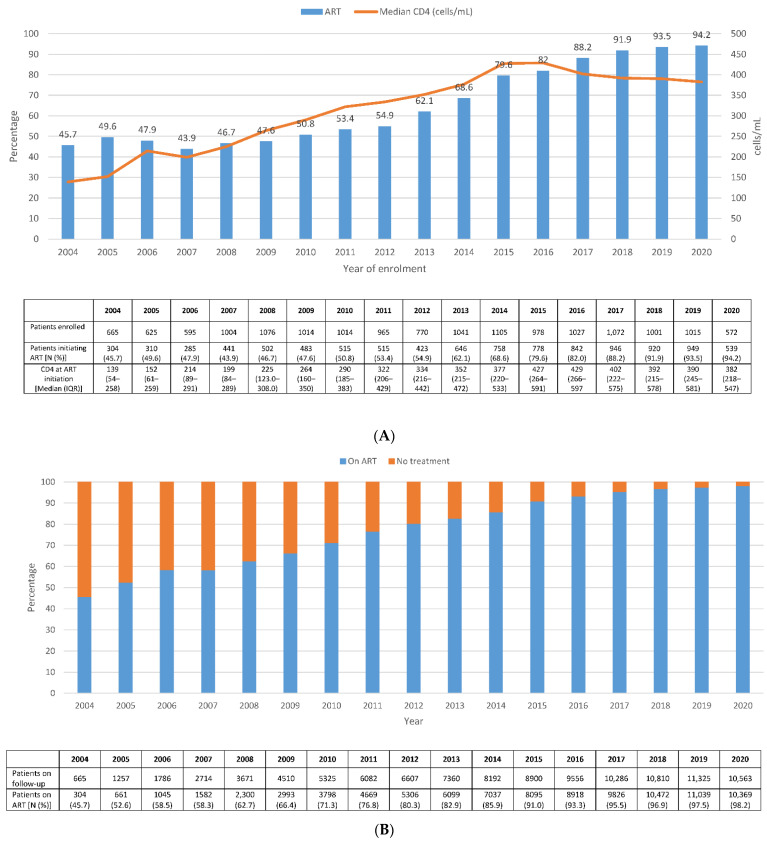
Temporal trends in the percentage of (**A**) patients initiating ART according to the year of enrollment in CoRIS and (**B**) patients on ART treatment, 2004–2020.

The percentage of patients who started treatment during the same year of their inclusion in the cohort remained roughly stable around 45.7% in 2004 to 54.9% in 2012, while since 2013, it started to increase up to 94.2% in 2020. Median CD4 count at ART initiation increased from 139 (IQR: 54–258) cells/µL in 2004 to 382.5 (IQR: 218–547.5) cells/µL in 2020. Although median CD4 cells at ART initiation decreased slightly from 2016 to 2020, this trend was not statistically significant (Figure 1A).

**Figure 2:**
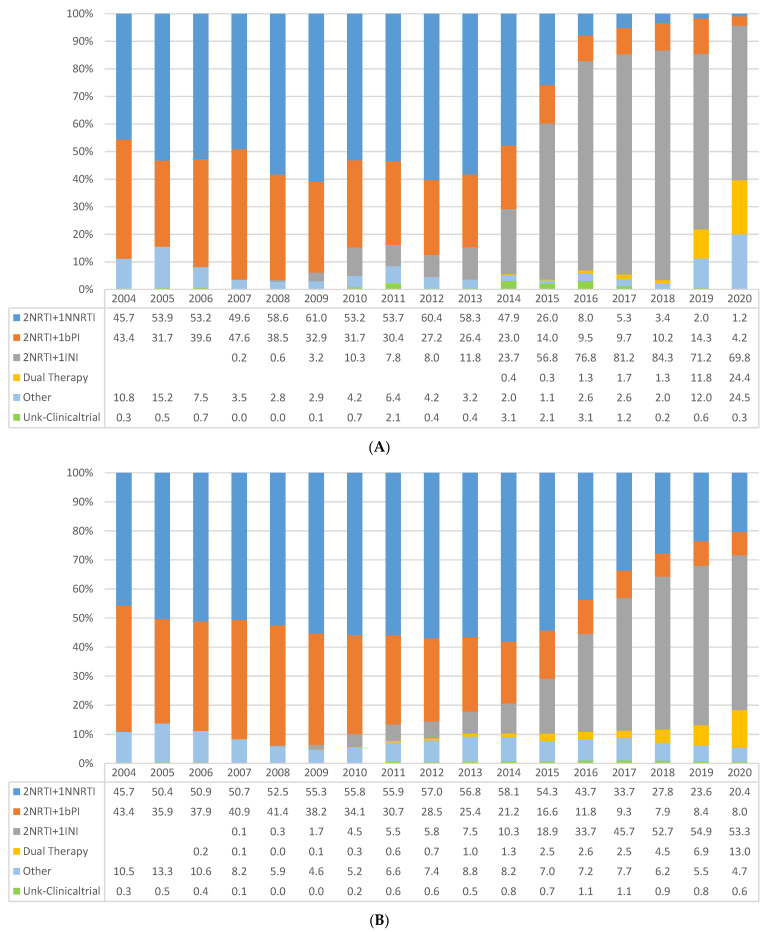
Temporal trends in the prescription of (**A**) first-line antiretroviral regimen among treatment-naïve patients and (Dual therapies as initial treatment were DTG/3TC and ETR + RAL). (**B**) ART regimen among all patients on treatment, by type of regimen, 2004–2020.(Dual therapies among patients on treatment were DTG/RPV, DTG/3TC, DRV/r + 3TC, DRV/r + RPV, DRV/r + ETV, DRV/r + DTG, DRV/r + RAL, CAB + RPV, ATV/r + 3TC, ETR + RAL, and DRVc + 3TC).

Figure 2A shows the proportions of initial treatment regimens prescribed in ART-naïve patients each year. The use of 2NRTI plus 1NNRTI (which were the most frequently prescribed regimens until 2014) and 2NRTI plus 1bPI decreased steadily after 2014, being gradually replaced by INI-based triple therapies. Since 2019, other regimens started to be prescribed, mainly dual therapies.

Since its authorization in 2007, TDF/FTC/EFV was the more frequently prescribed single-tablet regimen (STR) as initial ART, reaching 53.9% of all initial prescriptions in 2012, but experienced a decrease thereafter, as TDF/FTC/RPV, TDF/FTC/EVG/COBI, and ABC/3TC/DTG became successively available. TAF/FTC/BIC, which was marketed in 2018, accounted for 53.6% of the initial prescriptions in 2020, followed by the dual therapy DTG/3TC (24.0%) (Table S2).

**Figure 3:**
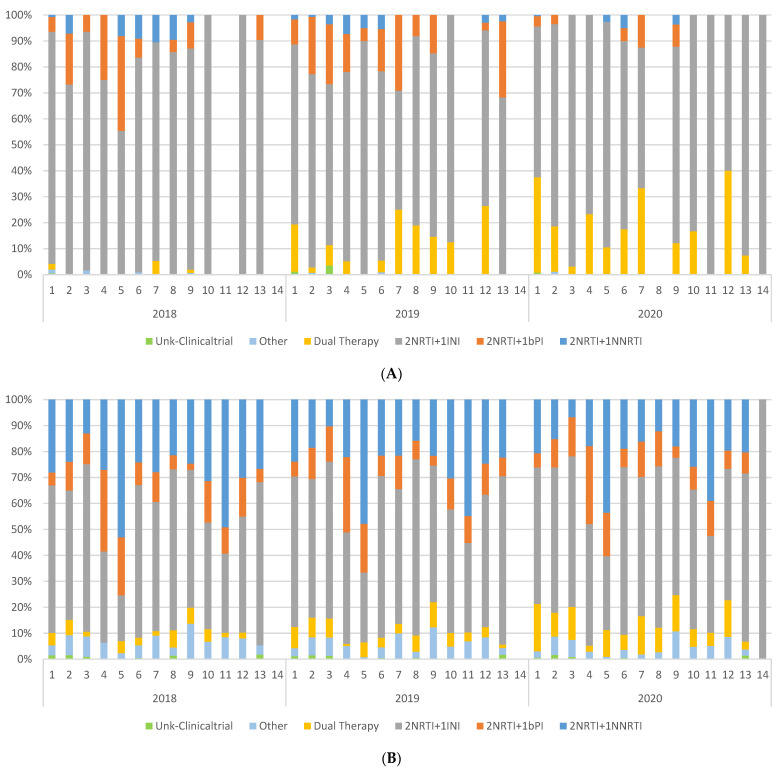
Geographic variability among autonomous regions in the prescription of (**A**) first-line antiretroviral regimen among treatment-naïve patients and (Dual therapies prescribed as initial treatment were DTG/3TC and ETR + RAL. Patients were not enrolled in the cohort in AR 11 in 2018 and 2019, or in AR 8 in 2020. AR 14 was included in the cohort in 2020). (**B**) ART regimen among all patients on treatment, by type of regimen, 2018–2020. (Dual therapies prescribed among patients on treatment were DTG/RPV, DTG/3TC, DRV/r + 3TC, DRV/r + RPV, DRV/r + ETV, DRV/r + DTG, DRV/r + RAL, CAB + RPV, ATV/r + 3TC, ETR + RAL, and DRVc + 3TC. AR 14 was included in the cohort in 2020). Each autonomous region (AR) is represented with a number.

As shown in Figure 3A, there were differences in the prescriptions of first-line antiretroviral regimen by autonomous region. In 2020, the percentage of patients initiating with 2NRTI plus 1INI ranged from 54.2% to 100%, whereas between 0% and 40% of patients started ART with other regimens, mainly dual therapies. During this same year, the percentage of patients initiating with 2NRTI plus 1bPI and 2NRTI plus 1NNRTI remained low (below 12.5% and 5%, respectively), in all regions.

The percentage of patients on ART among all those on active follow-up increased steadily over the years, from 45.7% in 2004 to 98.2% in 2020 (Figure 1B). Among all patients receiving ART, regimens based on 2NRTI plus 1INI steadily increased from 0.1% in 2007 to 53.3% in 2020. Regimens based on 2NRTI plus 1NNRTI were still administered to over 20% of patients in 2020 (Figure 2B). During 2007–2015, most patients on treatment were receiving TDF/FTC/EFV (used by up to 43.2% of the patients in 2012), which was replaced from 2017 onwards by ABC/3TC/DTG (used by 20–25% of the patients after this year). Prescription of dual therapies increased in recent years: in 2020, 8.8% of the patients were receiving DTG/3TC, and 2.3% were receiving DTG/RPV (Table S3).

There were also variations by autonomous region in the regimens that all patients on active follow-up were receiving (Figure 3B). In recent years, 2NRTI plus 1NNRTI regimens were replaced by 2NRTI plus INI and dual therapies. The uptake of dual therapies had high variability among autonomous regions (Figure 3B).

## 4. Discussion

In this large multicenter cohort in Spain, we have shown real-world data on temporal trends during a 17-year period and geographic variability in the use of ART and prescribed regimens in more than 15,000 individuals. Prior studies have assessed trends in ART prescription, but they covered shorter periods of time and none of them provided data at a national level [3,12]. To our knowledge, this is the first study assessing trends in ART over the whole life of a large national HIV cohort considering initial prescriptions as well as all patients on treatment, and geographic variability.

Since 2015, European and Spanish HIV treatment guidelines have recommended early ART initiation at diagnosis, regardless of immune status, in order to prevent HIV-related morbidity, mortality, and to reduce onward transmission [13,14]. This was reflected in our results, where more than 80% of the patients started treatment the same year of their enrollment since 2015. Moreover, from 2015 onwards, more than 90% of all the patients in the cohort were on ART, thus reaching the second “90” of the “90-90-90” target of the Joint United Nations Programme on HIV/AIDS for 2020 [15]. Interestingly, our analysis shows an upward trend before 2015 when it was recommended to start with progressively higher CD4 count.

In the European Union, the first STR was introduced in 2007, and to date, there are more than 10 approved STR options (Table S4). In our study, in most years, 40–50% of patients had an STR as initial treatment, showing compliance with the Spanish clinical guidelines that recommend treatment with an STR as the first option whenever possible [13,16]. For a long period, the only available STR was TDF/FTC/EFV, and the side effects of this regimen may have been more acceptable to patients and providers in the absence of other simple regimens [3]. The declining preference of TDF/FTC/EFV as a first-line treatment over time was probably due not only to the availability of other STRs but also to the change in the national guidelines’ recommendations, as since 2015, the only preferred regimens were those based on INI, and all the other regimens were considered as alternatives [17]. Since INI-based therapies became the only preferred ARTs in 2015, this was quickly reflected in our cohort, where the prescription of the previously preferred NNRTI-based regimens became minimal (less than 10%) among ART-naïve patients after that year. However, it is worth noting that among all the patients in the cohort, a considerable proportion of them were still receiving NNRTI-based regimens after 2015 (these regimens still being used by 20% of the patients in 2020), which shows that even though they were relegated as a second option in the guidelines, clinicians tend to maintain them in virally suppressed patients if the tolerance is acceptable.

In recent years, regimens based on INI have emerged as the most commonly prescribed first-line ART. In this regard, TAF/FTC/BIC was included in national guidelines’ recommendations for naïve HIV-positive individuals in 2019 and since then, this combination has rapidly relegated others as the most prescribed first-line ART. Dual therapy with DTG + 3TC was first used in 2016. Its prescription was only anecdotal until 2019, when it steeply increased to reach almost one quarter of all initial treatments in 2020; this was probably due to its introduction as STR and its recommendation as initial therapy in the national clinical guidelines, both occurring in 2019 [18]. In this regard, our contemporary cohort of patients shows how recent changes in clinical guidelines and availability of newer drugs or drug presentations are quickly translated into clinical practice [16,19,20]. We have not been able to analyze newer regimens such as cabotegravir + rilpivirine, as this treatment was still not marketed in Spain during the study period, and therefore, it was not used in our cohort outside of clinical trials.

Overall, treatment regimens were prescribed differently across autonomous regions; this was more evident for the ART regimens prescribed to all patients in the cohort than to treatment-naïve patients. This is in line with previous studies in this cohort that showed that the choice of initial ARV regimens varied significantly by hospital location [6]. Geographic variability in health care practices has been well described in several conditions, such as osteoporosis treatment, or urologic cancer [21,22]. Possible contributors to geographical variation in our study include policies of different autonomous regions and different hospital practices and physicians’ preferences, among others. Regarding the latter, the Spanish AIDS Study Group (GeSIDA) issues annual clinical guidelines that are widely known among clinicians caring for PLWHIV; these clinicians are hospital-based specialists with proper knowledge of the latest guideline recommendations, which are widely followed for initial ART in around 90% of patients [8,23]. Some restrictions, however, might have had an influence on the treatments prescribed in different autonomous regions: we have previously described that during the period 2010–2015, 54.1% of the centers in our cohort had a cost limitation for the prescription of ART and 29.7% had restricted access to at least one antiretroviral or STR (mainly some of the newest INI) [8,24]. While we have not formally assessed these limitations in this study, it is possible that they could partly explain our findings.

Our study of the geographic variability is limited by the fact that the names of each autonomous region cannot be disclosed due to confidentiality reasons. Moreover, the sample size and number of participating hospitals within each geographic unit may affect the accuracy of the analysis and the interpretation of variations among autonomous regions. In addition, the analysis is not adjusted/weighted on the number of people living with HIV in each region.

Substantial disruptions in HIV care after the start of the COVID-19 pandemic have been reported worldwide. National consequences are still uncertain, and it will take some time to ascertain how the COVID-19 pandemic has affected the diagnosis of new HIV infections and ARV provision. However, within the CORIS cohort, ARV delivery models were rapidly designed and successfully implemented to meet emergency needs brought on by the pandemic.

Our study has several strengths. It includes novel ART and newly authorized STRs; due to their novelty, many of these contemporary ART regimens have not been studied in observational cohorts. It is a large multicenter cohort with a reasonably large sample size and strict quality control procedures, and is largely representative of the newly diagnosed PLHIV in the Spanish general population [25,26]. Most standard clinical trials do not investigate treatment changes within the same trial but tend to focus on one medication, whereas observational studies performed with large databases allow the investigation of prescription trends of drugs manufactured by different pharmaceutical companies [27]. Modern ARTs have led to a reduction in the pill burden and simplified therapies such as mono/dual therapies or long-acting agents. In addition, there are multiple promising approaches currently in research and development to achieve a functional cure. This study allows us to better understand the real-world use of more than 15 years of ART, including the contemporary treatment patterns. To our knowledge, there are no data in the literature at a national level illustrating the temporal trends in ART prescription and geographical variation in Spain.

## 5. Conclusions

In conclusion, in this study, we evaluated the trends in ART prescription among treatment-naïve and -experienced PLWHIV in a real-world, multicenter national cohort from 2004 to 2020. Our findings show how temporal trends in ART prescription vary in clinical practice and reflect the availability of new ARVs and the changing guidelines’ recommendations. The geographic variability in ART prescription deserves further evaluation.

## Data Availability

Data are contained within the article or Supplementary Materials.

## Supplementary Materials

The following supporting information can be downloaded at: https://www.mdpi.com/article/10.3390/jcm11071896/s1, Table S1: Patients’ characteristics at ART initiation according to the period of ART initiation. Table S2: Temporal trends in the prescription of the first-line antiretroviral regimen, 2004–2020. Table S3: Temporal trends in the ART regimens received among all patients on treatment, 2004–2020. Table S4: Marketing dates of selected antiretroviral drugs in Spain (January 2022). List of CoRIS participating centers.
