# Supplementary material for: Temporal Trends and Geographic Variability in the Prescription of Antiretroviral Treatments in People Living with HIV in Spain, 2004–2020
Source: J Clin Med. 2022 Mar 29;11(7):1896. doi: 10.3390/jcm11071896 (PMC8999235; doi:10.3390/jcm11071896)
Supplement: Supplementary file 1 [file jcm-11-01896-s001.zip › jcm-1611399-supplementary.pdf]

Supplementary Material

Table S1. Patients' characteristics at ART initiation according to the period of ART initiation.

	2004-2007<br>n= 1,712	2008-2011<br>n= 3,566	2012-2015<br>n= 4,266	2016-2020<br>n= 5,074	*p*-value
**Sex at birth; n (%)**					
Male	1,296 (75.7)	2,900 (81.3)	3,743 (87.7)	4,453 (87.8)	<0.001
Female	416 (24.3)	666 (18.7)	523 (12.3)	621 (12.2)	
**Age at ART initiation (years); n (%)**					
<30	341 (19.9)	816 (22.9)	1,086 (25.5)	1,469 (28.9)	<0.001
30-40	657 (38.4)	1,368 (38.4)	1,587(37.2)	1,734 (34.2)	
>40	714 (41.7)	1,382 (38.7)	1,593 (37.3)	1,871 (36.9)	
Median (Q1, Q3)	38.1 (31.5, 44.6)	37.0 (30.6,44.3)	36.3 (29.8, 43.9)	35.8 (28.9,44.5)	
**Education level; n (%)**					
Elementary studies	104 (6.0)	123 (3.4)	91 (2.1)	103 (2.0)	
Primary	201 (11.7)	357 (10.0)	450 (10.5)	488 (9.6)	
Secondary	403 (23.5)	660 (18.5)	679 (15.9)	559 (11.0)	<0.001
High School	458 (26.7)	1,000 (28.0)	1,175 (27.5)	1,430 (28.2)	
Upper education	238 (13.9)	840 (23.6)	1,151 (26,9)	1,362 (26.8)	
Others	37 (2.2)	63 (1.8)	74 (1.7)	65 (1.3)	
Unknown	271 (15.8)	523 (14.7)	646 (15.1)	1,067 (21.0)	
**HIV transmission mode; n (%)**					
Men sex with men	610 (35.6)	1,955 (54.8)	2,867 (67.2)	3,404 (67.1)	<0.001
Heterosexual	730 (42.6)	1,187 (33.3)	1,059 (24.8)	1,273 (25.1)	
Injection drug use	312 (18.2)	316 (8.9)	172 (4.0)	124 (2.4)	
Other/Unknown	60 (3.5)	108 (3.0)	168 (3.9)	273 (5.4)	
**Clinical AIDS at ART initiation; n (%)**					

No<br>Yes	1,682 (98.2)<br>30 (1.8)	3,508 (98.4)<br>58 (1.6)	4,215 (98.8)<br>51 (1.2)	5,007 (98.7)<br>67(1.3)	0.209
**Region of birth; n (%)**					
Spain	1,039 (60.7)	2,075 (58.2)	2,633 (61.7)	2,803 (55.2)	
Latin America	269 (15.7)	666 (18.7)	735 (17.2)	1,416 (27.9)	
Western Europe	221 (12.9)	469 (13.1)	588 (13.8)	407 (8.0)	<0.001
Eastern Europe	27 (1.6)	94 (2.6)	82 (1.9)	99 (1.9)	
Sub-Saharan Africa	127 (7.4)	201 (5.6)	139 (3.3)	210 (4.1)	
Northern Africa	24 (1.4)	45 (1.3)	35 (0.8)	74 (1.4)	
Other	3 (0.2)	12 (0.3)	28 (0.7)	32 (0.6)	
Unknown	2 (0.1)	4 (0.1)	26 (0.6)	33 (0.6)	
**Autonomous regions**; n (%)**					
1	724 (42.3)	1,421 (39.8)	1,441 (33.8)	1,951 (38.5)	
2	195 (11.4)	397 (11.1)	452 (10.6)	564 (11.1)	
3	0 (0.0)	2 (0.1)	70 (1.6)	190 (3.7)	
4	10 (0.6)	74 (2.1)	52 (1.2)	47 (0.9)	
5	133 (7.7)	284 (7.9)	354 (8.3)	329 (6.5)	
6	123 (7.2)	269 (7.5)	454 (10.6)	485 (9.6)	<0.001
7	65 (3.8)	130 (3.6)	83 (1.9)	134 (2.6)	
8	0 (0.0)	82 (2.3)	129 (3.0)	103 (2.0)	
9	255 (14.9)	571 (16.0)	796 (18.7)	769 (15.2)	
10	71 (4.1)	75 (2.1)	68 (1.6)	71 (1.4)	
11	14 (0.8)	38 (1.1)	22 (0.5)	9 (0.2)	
12	48 (2.8)	76 (2.1)	140 (3.3)	152 (3.0)	
13	74 (4.3)	147 (4.1)	205 (4.8)	267 (5.3)	
14	0 (0.0)	0 (0.0)	0 (0.0)	3 (0.1)	

*Abbreviations: Q1, 1st quartile; Q3, 3rd quartile.
** The names of the AR cannot be disclosed due to confidentiality reasons.

Table S2. Temporal trends in the prescription of the first-line antiretroviral regimen, 2004-2020

2004			2005			2006			2007			2008			2009		
**ART**	**N**	**%**	**ART**	**N**	**%**	**ART**	**N**	**%**	**ART**	**N**	**%**	**ART**	**N**	**%**	**ART**	**N**	**%**
AZT/3TC+EFV	55	18.1	TDF+3TC+EFV	65	17.0	TDF/FTC+EFV	121	28.3	TDF/FTC/EFV	198	33.1	TDF/FTC/EFV	332	42.3	TDF/FTC/EFV	435	52.9
AZT/3TC+LPV+RTV	46	15.1	AZT/3TC+EFV	53	13.9	TDF/FTC+LPV+RTV	50	11.7	TDF/FTC+LPV+RTV	118	19.7	TDF/FTC+LPV/R	154	19.6	TDF/FTC+LPV/R	79	9.6
ddI+3TC+EFV	35	11.5	AZT/3TC+LPV+RTV	40	10.5	ABC/3TC+EFV	29	6.8	ABC/3TC+EFV	42	7.0	TDF/FTC+NVP	48	6.1	TDF/FTC+ATV+RTV	74	9.0
TDF+3TC+EFV	22	7.2	ddI+3TC+EFV	32	8.4	AZT/3TC+EFV	19	4.5	ABC/3TC+LPV+RTV	42	7.0	ABC/3TC+EFV	44	5.6	TDF/FTC+DRV+RTV	46	5.6
TDF+3TC LPV+RTV	19	6.3	TDF/FTC +EFV	19	5.0	AZT/3TC+LPV+RTV	19	4.5	TDF/FTC+RTV+FPV	32	5.3	TDF/FTC+FPV+RTV	33	4.2	TDF/FTC+NVP	26	3.2
ABC/3TC+LPV+RTV	14	4.6	ABC/3TC+AZT	17	4.5	TDF/FTC+NVP	19	4.5	TDF/FTC+NVP	29	4.8	TDF/FTC+ATV+RTV	29	3.7	ABC/3TC+EFV	20	2.4
ddI+3TC+LPV+RTV	14	4.6	AZT/3TC+NVP	17	4.5	ABC/3TC+ATV+RTV	15	3.5	AZT/3TC+LPV+RTV	20	3.3	AZT/3TC+LPV/R	28	3.6	AZT/3TC+LPV/R	18	2.2
AZT/3TC+NVP	13	4.3	ABC/3TC+EFV	13	3.4	TDF/FTC+RTV+SQV	14	3.3	TDF/FTC+RTV+SQV	18	3.0	ABC/3TC+LPV/R	23	2.9	TDF/FTC+RAL	16	1.9
ABC/3TC+AZT	12	4.0	TDF+3TC+ LPV+RTV	13	3.4	ABC/3TC+LPV+RTV	12	2.8	ABC/3TC+FPV+RTV	16	2.7	AZT/3TC+EFV	11	1.4	TDF/FTC+FPV+RTV	14	1.7
ABC/3TC+FPV+RTV	9	3.0	ABC/3TC+LPV+RTV	9	2.4	TDF+3TC+EFV	12	2.8	TDF/FTC+ATV+RTV	10	1.7	ddI+3TC+EFV	11	1.4	ABC/3TC+LPV/R	11	1.3
d4T+3TC+LPV+RTV	9	3.0	LPV+RTV+NVP	9	2.4	TDF/FTC+FPV+RTV	12	2.8	AZT/3TC+NVP	10	1.7	ABC/3TC+ATV+RTV	8	1.0	ABC/3TC+ATV+RTV	9	1.1
AZT/3TC+NFV+RTV	8	2.6	AZT/3TC+NFV+RTV	8	2.1	AZT/3TC+NVP	11	2.6	AZT/3TC+EFV	7	1.2	OTHER	64	8.2	ATV+RTV+MVC	9	1.1
d4T+3TC+EFV	8	2.6	TDF/FTC +LPV	8	2.1	ABC/3TC+AZT	10	2.3	AZT/3TC+RTV+SQV	7	1.2				OTHER	66	8.0
ABC/3TC+AZT+LPV+RTV	7	2.3	ABC/3TC+AZT+TDF	7	1.8	TDF/FTC+ATV+RTV	10	2.3	OTHER	50	8.3						
AZT/3TC+ABC+EFV	6	2.0	ABC/3TC+AZT+LPV+RTV	5	1.3	AZT/3TC+RTV+SQV	10	2.3									
TDF+3TC+TPV+RTV	5	1.6	AZT/3TC+FPV+RTV	5	1.3	ABC/3TC+NVP	8	1.9									
OTHER	22	7.2	AZT/3TC+RTV+SQV	5	1.3	ABC/3TC+RTV+SQV	7	1.6									
			d4T+3TC+LPV+RTV	5	1.3	ddI+3TC+EFV	5	1.2									
			ddI+3TC+LPV+RTV	5	1.3	OTHER	44	10.3									
			TDF+3TC+ FPV+RTV	4	1.1												
			LPV+RTV	4	1.1												
			TDF+3TC+NVP	4	1.1												
			OTHER	35	9.2												

2010			2011			2012			2013			2014			2015		
**ART**	**N**	**%**	**ART**	**N**	**%**	**ART**	**N**	**%**	**ART**	**N**	**%**	**ART**	**N**	**%**	**ART**	**N**	**%**
TDF/FTC/EFV	425	45.2	TDF/FTC/EFV	494	48.6	TDF/FTC/EFV	436	53.9	TDF/FTC/EFV	288	28.0	TDF/FTC/RPV	324	27.9	TDF/FTC/EVG/COBI	279	22.1
TDF/FTC+DRV+RTV	161	17.1	TDF/FTC+DRV+RTV	151	14.9	TDF/FTC+DRV+RTV	106	13.1	TDF/FTC/RPV	241	23.4	FTC/TDF/EFV	172	14.8	ABC/3TC/DTG	271	21.4
TDF/FTC+RAL	84	8.9	TDF/FTC+ATV+RTV	80	7.9	TDF/FTC+ATV+RTV	51	6.3	TDF/FTC+DRV+RTV	154	15.0	TDF/FTC+DRV+RTV	163	14.0	TDF/FTC/RPV	216	17.1
TDF/FTC+ATV+RTV	57	6.1	TDF/FTC+RAL	35	3.4	TDF/FTC+RAL	36	4.5	TDF/FTC+RAL	76	7.4	TDF/FTC+RAL	113	9.7	TDF/FTC+DRV+RTV	112	8.9
TDF/FTC+LPV/R	38	4.0	ABC/3TC/DTG	32	3.2	TDF/FTC+LPV/R	24	3.0	TDF/FTC+ATV+RTV	40	3.9	TDF/FTC/EVG/COBI	72	6.2	TDF/FTC+DTG	97	7.7
TDF/FTC+NVP	29	3.1	TDF/FTC+LPV/R	32	3.2	ABC/3TC+EFV	14	1.7	ABC/3TC+ATV+RTV	21	2.0	TDF/FTC+ATV+RTV	41	3.5	TDF/FTC/EFV	57	4.5
ABC/3TC+NVP	19	2.0	Clinical Trial	21	2.1	DRV+RTV+MVC	13	1.6	ABC/3TC+RAL	21	2.0	ABC/3TC+DRV+RTV	39	3.4	TDF/FTC+RAL	50	4.0
ABC/3TC+EFV	15	1.6	TDF/FTC+NVP	20	2.0	ABC/3TC+ATV+RTV	12	1.5	ABC/3TC+DRV+RTV	19	1.8	ABC/3TC/DTG	38	3.3	Clinical Trial	27	2.1
ABC/3TC+ATV+RTV	13	1.4	ABC/3TC+DRV+RTV	19	1.9	ABC/3TC+DRV+RTV	12	1.5	TDF/FTC+NVP	19	1.8	Clinical Trial	36	3.1	ABC/3TC+RAL	20	1.6
ATV+RTV+MVC	11	1.2	ATV+RTV+MVC	18	1.8	TDF/FTC+ETR	12	1.5	ABC/3TC+EFV	16	1.6	ABC/3TC+RAL	36	3.1	ABC/3TC+DRV+RTV	17	1.3
AZT/3TC+LPV/R	10	1.1	ABC/3TC+ATV+RTV	12	1.2	ABC/3TC+NVP	11	1.4	ABC/3TC+NVP	16	1.6	ABC/3TC+RPV	36	3.1	ABC/3TC+RPV	17	1.3
OTHER	79	8.4	OTHER	103	10.1	TDF/FTC+NVP	11	1.4	TDF/FTC/EVG/COBI	15	1.5	ABC/3TC+EFV	14	1.2	ABC/3TC+EFV	16	1.3
						ABC/3TC+RAL	10	1.2	TDF/FTC+LPV/R	15	1.5	ABC/3TC+ATV+RTV	13	1.1	TDF/FTC+DRV/COBI	16	1.3
						ABC/3TC+DTG	9	1.1	ABC/3TC+LPV/R	12	1.2	ABC/3TC+CAB	12	1.0	TDF/FTC+DOR	16	1.3
						TDF/FTC+DTG	9	1.1	OTHER	77	7.5	OTHER	54	4.6	TDF/FTC+ATV+RTV	15	1.2
						OTHER	43	5.3							OTHER	38	3.0

2016			2017			2018			2019			2020		
**ART**	**N**	**%**	**ART**	**N**	**%**	**ART**	**N**	**%**	**ART**	**N**	**%**	**ART**	**N**	**%**
ABC/3TC/DTG	426	37.0	ABC/3TC/DTG	412	34.1	ABC/3TC/DTG	322	30.0	TAF/FTC/BIC	335	32.1	TAF/FTC/BIC	319	53.6
TAF/FTC/EVG/COBI	153	13.3	TAF/FTC/EVG/COBI	328	27.1	TAF/FTC/EVG/COBI	260	24.2	ABC/3TC/DTG	177	16.9	DTG/3TC	143	24.0
TDF/FTC/EVG/COBI	137	11.9	TDF/FTC+DTG	154	12.7	TDF/FTC+DTG	171	15.9	TAF/FTC/DRV/COBI	144	13.8	TDF/FTC+DTG	43	7.2
TDF/FTC+DTG	111	9.6	TDF/FTC+DRV/COBI	47	3.9	TAF/FTC+DTG	83	7.7	DTG/3TC	123	11.8	TAF/FTC/DRV/COBI	25	4.2
TDF/FTC/RPV	71	6.2	TDF/FTC+RAL	47	3.9	TAF/FTC/DRV/COBI	67	6.2	TDF/FTC+DTG	114	10.9	ABC/3TC/DTG	22	3.7
TDF/FTC+DRV/COBI	40	3.5	TDF/FTC/RPV	45	3.7	TDF/FTC+RAL	45	4.2	TAF/FTC/EVG/COBI	51	4.9	TDF/FTC+RAL	13	2.2
TDF/FTC+DRV+RTV	37	3.2	TDF/FTC+DRV+RTV	30	2.5	TDF/FTC+DRV+RTV	23	2.1	TAF/FTC+DTG	30	2.9	TAF/FTC+DTG	9	1.5
Clinical Trial	36	3.1	DTG+3TC	21	1.7	TAF/FTC+RAL	19	1.8	TDF/FTC+RAL	24	2.3	OTHER	21	3.5
TDF/FTC+RAL	36	3.1	TAF/FTC/DRV/COBI	16	1.3	TDF/FTC/RPV	18	1.7	OTHER	47	4.5			
TDF/FTC/EFV	15	1.3	TAF/FTC+DTG	16	1.3	TDF/FTC+DRV/COBI	14	1.3						
TAF/FTC/DRV/COBI	14	1.2	Clinical Trial	15	1.2	TAF/FTC/RPV	14	1.3						
DTG+3TC	13	1.1	OTHER	78	6.5	DTG+3TC	12	1.1						
OTHER	63	5.5				OTHER	25	2.3						

Table S3. Temporal trends in the ART regimens received among all patients on treatment, 2004-2020

2004			2005			2006			2007			2008			2009		
ART	N	%	ART	N	%	ART	N	%	ART	N	%	ART	N	%	ART	N	%
AZT/3TC+EFV	55	18.1	AZT/3TC+EFV	93	14.1	TDF/FTC +EFV	155	14.8	TDF/FTC/EFV	397	25.1	TDF/FTC/EFV	737	32.0	TDF/FTC/EFV	1,184	39.6
AZT/3TC +LPV+RTV	46	15.1	TDF+3TC+EFV	93	14.1	AZT/3TC+EFV	97	9.3	TDF/FTC+LPV+RTV	198	12.5	TDF/FTC+LPV/R	381	16.6	TDF/FTC+LPV/R	415	13.9
ddI+3TC+EFV	35	11.5	AZT/3TC+LPV+RTV	74	11.2	TDF+3TC+EFV	84	8.0	ABC/3TC+EFV	87	5.5	ABC/3TC+EFV	121	5.3	TDF/FTC+ATV+RTV	177	5.9
TDF+3TC+EFV	22	7.2	ddI+3TC+EFV	62	9.4	AZT/3TC+LPV+RTV	69	6.6	AZT/3TC+EFV	72	4.6	TDF/FTC+NVP	116	5.0	TDF/FTC+NVP	143	4.8
TDF+3TC+ LPV+RTV+	19	6.3	ABC/3TC+AZT	35	5.3	TDF/FTC+LPV+RTV	65	6.2	ABC/3TC+LPV+RTV	70	4.4	TDF/FTC+FPV+RTV	87	3.8	ABC/3TC+EFV	137	4.6
ABC/3TC+LPV+RTV	14	4.6	TDF+3TC+LPV+RTV	33	5.0	ddI+3TC+EFV	63	6.0	TDF/FTC +NVP	62	3.9	ABC/3TC+LPV/R	86	3.7	TDF/FTC+FPV+RTV	95	3.2
ddI+3TC+LPV+RTV	14	4.6	AZT/3TC+NVP	31	4.7	ABC/3TC+AZT	51	4.9	TDF/FTC+FPV+RTV	59	3.7	TDF/FTC+ATV+RTV	78	3.4	ABC/3TC+LPV/R	92	3.1
AZT/3TC+NVP	13	4.3	ABC/3TC+LPV+RTV	22	3.3	ABC/3TC+EFV	46	4.4	TDF+3TC+EFV	58	3.7	AZT/3TC+LPV/R	63	2.7	TDF/FTC+DRV+RTV	56	1.9
ABC/3TC+AZT	12	4.0	TDF/FTC +EFV	21	3.2	AZT/3TC+NVP	37	3.5	AZT/3TC+LPV+RTV	57	3.6	AZT/3TC+EFV	62	2.7	AZT/3TC+LPV/R	54	1.8
ABC/3TC+FPV+RTV	9	3.0	ddI+3TC+LPV+RTV	14	2.1	ABC/3TC+LPV+RTV	35	3.4	ddI+3TC+EFV	49	3.1	TDF+3TC+EFV	45	2.0	ABC/3TC+ATV+RTV	53	1.8
d4T+3TC+LPV+RTV	9	3.0	ABC/3TC+EFV	13	2.0	TDF+3TC+LPV+RTV	27	2.6	ABC/3TC+AZT	48	3.0	ABC/3TC+AZT	44	1.9	ABC/3TC+NVP	51	1.7
AZT/3TC+NFV+RTV	8	2.6	AZT/3TC+NFV+RTV	13	2.0	TDF/FTC +NVP	23	2.2	AZT/3TC+NVP	41	2.6	ddI+3TC+EFV	42	1.8	TDF/FTC+RAL	38	1.3
d4T+3TC+EFV	8	2.6	d4T+3TC+LPV+RTV	13	2.0	TDF/FTC+FPV+RTV	19	1.8	TDF/FTC+SQV+RTV	39	2.5	TDF/FTC+SQV+RTV	41	1.8	TDF/FTC+SQV+RTV	37	1.2
ABC/3TC+AZT+LPV+RTV	7	2.3	ABC/3TC+FPV+RTV	11	1.7	ABC/3TC+ATV+RTV	16	1.5	TDF/FTC+ATV+RTV	35	2.2	ABC/3TC+ATV+RTV	38	1.7	AZT/3TC+EFV	30	1.0
AZT/3TC+ABC+EFV	6	2.0	TDF/FTC+LPV+RTV	10	1.5	AZT/3TC+SQV+RTV	16	1.5	ABC/3TC+FPV+RTV	27	1.7	AZT/3TC+NVP	32	1.4	OTHER	431	14.4
TDF+3TC+TPV+RTV	5	1.6	ABC/3TC+AZT+LPV+RTV	8	1.2	TDF/FTC+SQV+RTV	16	1.5	ABC/3TC+ATV+RTV	26	1.6	ABC/3TC+NVP	30	1.3			
OTHER	22	7.2	OTHER	115	17.5	TDF+3TC+ ATV+RTV	15	1.4	ABC/3TC+NVP	21	1.3	ABC/3TC+FPV+RTV	29	1.3			
						ABC/3TC+FPV+RTV	14	1.3	AZT/3TC+SQV+RTV	19	1.2	OTHER	1.005	43.7			
						ABC/3TC+NVP	11	1.1	ABC/3TC+SQV+RTV	16	1.0						
						ABC/3TC+SQV+RTV	11	1.1	OTHER	201	12.7						
						ddI+3TC+LPV+RTV	11	1.1									
						OTHER	164	15.7									

2010			2011			2012			2013			2014			2015		
**ART**	**N**	**%**	**ART**	**N**	**%**	**ART**	**N**	**%**	**ART**	**N**	**%**	**ART**	**N**	**%**	**ART**	**N**	**%**
TDF/FTC/EFV	1570	41.3	TDF/FTC/EFV	1975	42.3	TDF/FTC/EFV	2,293	43.2	TDF/FTC/EFV	2,371	38.9	TDF/FTC/EFV	2,006	28.5	TDF/FTC/EFV	1751	21.6
TDF/FTC+LPV/R	379	10.0	TDF/FTC+DRV+RTV	426	9.1	TDF/FTC+DRV+RTV	517	9.7	TDF/FTC+DRV+RTV	598	9.8	TDF/FTC/RPV	1,219	17.3	TDF/FTC/RPV	1,661	20.5
TDF/FTC+DRV+RTV	251	6.6	TDF/FTC+LPV/R	322	6.9	TDF/FTC+ATV+RTV	327	6.2	TDF/FTC+ATV+RTV	329	5.4	TDF/FTC+DRV+RTV	632	9.0	TDF/FTC+DRV+RTV	600	7.4
TDF/FTC+ATV+RTV	233	6.1	TDF/FTC+ATV+RTV	282	6.0	TDF/FTC+LPV/R	282	5.3	TDF/FTC+RAL	267	4.4	TDF/FTC+RAL	340	4.8	TDF/FTC/EVG/COBI	459	5.7
TDF/FTC+NVP	195	5.1	TDF/FTC+NVP	231	5.0	TDF/FTC+NVP	230	4.3	TDF/FTC/RPV	262	4.3	TDF/FTC+ATV+RTV	292	4.2	ABC/3TC/DTG	421	5.2
TDF/FTC+RAL	140	3.7	TDF/FTC+RAL	182	3.9	TDF/FTC+RAL	198	3.7	TDF/FTC+NVP	239	3.9	TDF/FTC+NVP	200	2.8	TDF/FTC+RAL	316	3.9
ABC/3TC+EFV	125	3.3	ABC/3TC+EFV	130	2.8	ABC/3TC+EFV	140	2.6	TDF/FTC+LPV/R	205	3.4	ABC/3TC+NVP	162	2.3	ABC/3TC+RPV	254	3.1
ABC/3TC+NVP	95	2.5	ABC/3TC+NVP	112	2.4	TDF/FTC+ETR	127	2.4	TDF/FTC+ETR	160	2.6	ABC/3TC+DRV+RTV	159	2.3	TDF/FTC+ATV+RTV	220	2.7
TDF/FTC+FPV+RTV	86	2.3	ABC/3TC+ATV+RTV	91	2.0	ABC/3TC+NVP	124	2.3	ABC/3TC+EFV	159	2.6	ABC/3TC+EFV	157	2.2	ABC/3TC+EFV	188	2.3
ABC/3TC+ATV+RTV	83	2.2	TDF/FTC+ETR	67	1.4	ABC/3TC+ATV+RTV	99	1.9	ABC/3TC+NVP	148	2.4	TDF/FTC+LPV/R	149	2.1	ABC/3TC+RAL	180	2.2
ABC/3TC+LPV/R	65	1.7	TDF/FTC+FPV+RTV	66	1.4	ABC/3TC+DRV+RTV	82	1.6	ABC/3TC+ATV+RTV	118	1.9	TDF/FTC+ETR	127	1.8	ABC/3TC+DRV+RTV	177	2.2
AZT/3TC+LPV/R	44	1.2	ABC/3TC+DRV+RTV	61	1.3	ABC/3TC+LPV/R	61	1.2	ABC/3TC+DRV+RTV	113	1.9	ABC/3TC+ATV+RTV	120	1.7	TDF/FTC+NVP	164	2.0
OTHER	532	14.0	ABC/3TC+LPV/R	55	1.2	TDF/FTC+FPV+RTV	56	1.1	ABC/3TC+ETR	79	1.3	ABC/3TC+RAL	119	1.7	ABC/3TC+NVP	154	1.9
			OTHER	669	14.3	ABC/3TC+ETR	54	1.0	ABC/3TC+LPV/R	75	1.2	TDF/FTC/EVG/COBI	114	1.6	TDF/FTC+DTG	131	1.6
						OTHER	716	13.5	ABC/3TC+RAL	68	1.1	ABC/3TC+RPV	107	1.5	ABC/3TC+ATV+RTV	107	1.3
									OTHER	908	14.9	ABC/3TC/DTG	91	1.3	TDF/FTC+ LPV/R	103	1.3
												ABC/3TC+ETR	87	1.2	TDF/FTC+ETR	97	1.2
												OTHER	956	13.6	ABC/3TC+ETR	84	1.0
															OTHER	1,028	12.7

2016			2017			2018			2019			2020		
**ART**	**N**	**%**	**ART**	**N**	**%**	**ART**	**N**	**%**	**ART**	**N**	**%**	**ART**	**N**	**%**
TDF/FTC/RPV	1,689	18.9	ABC/3TC/DTG	2,206	22.5	ABC/3TC/DTG	2,647	25.3	ABC/3TC/DTG	2,838	25.7	ABC/3TC/DTG	2,241	21.6
ABC/3TC/DTG	1,390	15.6	TDF/FTC/RPV	1,619	16.5	TAF/FTC/EVG/COBI	1,811	17.3	TAF/FTC/EVG/COBI	1,799	16.3	TAF/FTC/BIC	1,810	17.5
TDF/FTC/EFV	1,325	14.9	TAF/FTC/EVG/COBI	1,190	12.1	TDF/FTC/RPV	1,463	14.0	TDF/FTC/RPV	902	8.2	DTG/3TC	916	8.8
TDF/FTC/EVG/COBI	745	8.4	TDF/FTC/EFV	959	9.8	TDF/FTC/EFV	723	6.9	TDF/FTC/EFV	551	5.0	TAF/FTC/RPV	885	8.5
TDF/FTC+DRV+RTV	394	4.4	TDF/FTC+DTG	315	3.2	TDF/FTC+DTG	353	3.4	TAF/FTC/RPV	547	5.0	TDF/FTC/EVG/COBI	761	7.3
ABC/3TC+RPV	318	3.6	TDF/FTC/EVG/COBI	297	3.0	ABC/3TC+RPV	309	3.0	TAF/FTC/DRV/COBI	505	4.6	TAF/FTC/DRV/COBI	550	5.3
TDF/FTC+RAL	286	3.2	ABC/3TC+RPV	291	3.0	TDF/FTC+RAL	227	2.2	DTG/3TC	404	3.7	TDF/FTC/EFV	412	4.0
TDF/FTC+DTG	233	2.6	TDF/FTC+RAL	269	2.7	TAF/FTC/DRV/COBI	209	2.0	TAF/FTC/BIC	377	3.4	TDF/FTC/RPV	339	3.3
TAF/FTC/EVG/COBI	172	1.9	TDF/FTC+COBI/DRV	245	2.5	TDF/FTC+DRV/COBI	187	1.8	TDF/FTC+DTG	344	3.1	DTG/RPV	243	2.3
ABC/3TC+RAL	155	1.7	TDF/FTC+DRV+RTV	243	2.5	DTG/RPV	171	1.6	ABC/3TC+RPV	289	2.6	ABC/3TC+RPV	228	2.2
ABC/3TC+EFV	142	1.6	DTG+RPV	175	1.8	TDF/FTC+DRV+RTV	169	1.6	TDF/FTC+RAL	204	1.9	TDF/FTC+DTG	221	2.1
ABC/3TC+NVP	139	1.6	ABC/3TC+RAL	161	1.6	ABC/3TC+RAL	162	1.6	DTG/RPV	178	1.6	DRV/COBI+3TC	159	1.5
TDF/FTC+DRV/COBI	137	1.5	ABC/3TC+NVP	118	1.2	DRV/COBI+3TC	158	1.5	ABC/3TC+RAL	175	1.6	TDF/FTC+RAL	155	1.5
TDF/FTC+NVP	135	1.5	TDF/FTC +NVP	111	1.1	TAF/FTC+DTG	149	1.4	TAF/FTC+DTG	174	1.6	ABC/3TC+RAL	138	1.3
ABC/3TC+DRV+RTV	131	1.5	ABC/3TC+EFV	104	1.1	DTG+3TC	122	1.2	DRV/COBI+3TC	171	1.6	CAB+RPV	109	1.1
TDF/FTC+ATV+RTV	128	1.4	Clinical Trial	103	1.1	OTHER	1,612	15.4	TDF/FTC+DRV+RTV	128	1.2	OTHER	1,202	12.0
DTG+RPV	108	1.2	OTHER	1,420	14.5				OTHER	1,453	13.2			
Clinical Trial	97	1.1												
OTHER	1,194	13.4												

Table S4. Marketing dates of selected antiretroviral drugs in Spain (January 2022)

Name	Date of authorisation	Brand name	Withdrawal date
Nucleoside/Nucleotide Reverse Transcriptase Inhibitors (NRTIs)			
Zidovudine (AZT,ZDV))	01/07/1988	Retrovir®	
Emtricitabine (FTC)	02/12/2003	Emtriva®	
Abacabir (ABC)	01/09/1999	Ziagen®	
Lamivudine (3TC)	01/11/1996	Epivir®	
Stavudine (d4T)	01/09/1996	Zerit®	31/08/2020
Didanosine (ddI)	16/08/2000	Videx®	17/01/2019
Tenofovir disoproxil fumarate (TDF)	25/02/2002	Viread®	
Tenofovir Alafenamide (TAF)	25/01/2017	Vemlidy®	
Zalcitabine (ddC)	01/03/1994	Hivid®	20/02/2007
NRTIs fixed dose combinations			
Abacavir/Lamivudine (ABC/3TC)	30/12/2004	Kivexa®	
Abacavir/Lamivudine/Zidovudine (ABC/3TC/AZT)	10/04/2014	Trizivir®	
Emtricitabine/Tenofovir disoproxil fumarate (FTC/TDF)	07/03/2005	Truvada®	
Emtricitabine/Tenofovir alafenamide (FTC/TAF)	12/05/2016	Descovy®	
Lamivudine/Zidovudine (FTC/AZT)	01/11/1998	Combivir®	

Non-Nucleoside Reverse Transcriptase Inhibitors (NNRTIs)			
Efavirenz (EFV)	01/07/1999	Sustiva®	
Etravirine (ETR)	16/12/2011	Intelence®	
Nevirapine (NVP)	01/04/1998	Viramune®	
Rilpivirine (RPV)	13/01/2012	Edurant®	
Doravirine (DOR)	08/02/2019	Pifeltro®	
Protease inhibitors			
Atazanavir (ATV)	29/03/2004	Reyataz®	
Darunavir (DRV)	16/02/2009	Prezista®	
Darunavir/Cobicistat (DRV/COBI)	26/12/2014	Rezolsta®	
Fosamprenavir (FPV)	22/07/2004	Telzir®	
Lopinavir/Ritonavir (LPV/r)	24/04/2008	Kaletra®	
Ritonavir (RTV)	15/10/2015	Norvir®	
Atazanavir/Cobiscitat (ATV/COBI)	30/07/2015	Evotaz®	
Saquinavir (SQV)	01/10/1996	Invirase®	30/08/2018
Indinavir (IDV)	01/11/1996	Crixivan®	06/04/2021
Tipranavir (TPV)	02/11/2005	Aptivus®	
Amprenavir (APV)	12/03/2001	Agenerase®	01/03/2008

Nelfinavir (NFV)	01/06/1998	Viracept®	13/02/2012
Integrase inhibitors			
Dolutegravir (DTG)	12/02/2014	Tivicay®	
Raltegravir (RAL)	21/12/2007	Isentress®	
Cabotegravir (CAB)	20/01/2021	Vocabria®	
CCR5 inhibitors			
Maraviroc (MVC)	27/09/2007	Celsentri®	
Fusion inhibitors			
Enfuvirtide (T-20)	05/06/2003	Fuzeon®	
Boosters			
Cobicistat (COBI)	28/03/2014	Tybost®	
Ritonavir (RTV)	01/10/1996	Norvir®	
Single dose régimen			
Efavirenz/Emtricitabine/ Tenofovir disoproxil (EFV/FTC/TDF)	21/12/2007	Atripla®	
Rilpirivina/Emtricitabine/Tenofovir disoproxil (RPV/FTC/TDF)	26/01/2012	Eviplera®	
Rilpirivina/Tenofovir alafenamide/ Emtricitabine (RPV/TAF/FTC)	13/07/2016	Odefsey®	

Elvitegravir/cobicistat/Emtricitabine/<br>Tenofovir alafenamide (EVT/COBI/FTC/TAF)	27/11/2015	Genvoya®
Elvitegravir/cobicistat/Emtricitabine/Tenofovir disoproxil (EVT/COBI/FTC/TDF)	26/06/2013	Stribild®
Dolutegravir/Abacavir/Lamivudine (DTG/ABC/3TC)	15/10/2014	Triumeq®
Dolutegravir/Rilpirivina (DTG/RPV)	27/06/2018	Juluca®
Bictegravir /Emtricitabine/Tenofovir alafenamide (BIC/FTC/TAF)	10/07/2018	Biktarvy®
Darunavir/cobicistat/Emtricitabine/<br>Tenofovir alafenamide (DRV/COBI/FTC/TAF)	27/10/2017	Symtuza®
Dolutegravir/Lamivudina (DTG/3TC)	19/07/2019	Dovato®

Origen: Bot Plus. Medicines Database of the General Council of Pharmaceutical Schools (BotPlus) (date access Jan 2022) and CIMA (AEMPS) (date access Jan 2022)

Centers and investigators involved in CoRIS cohort:

Executive committee: Santiago Moreno, Inma Jarrín, David Dalmau, Maria Luisa Navarro, Maria Isabel González, Federico Garcia, Eva Poveda, Jose Antonio Iribarren, Félix Gutiérrez, Rafael Rubio, Francesc Vidal, Juan Berenguer, Juan González, M Ángeles Muñoz-Fernández.

Fieldwork data management and analysis: Inmaculada Jarrin, Belén Alejos, Cristina Moreno, Carlos Iniesta, Marta Rava, Rebeca Izquierdo.

BioBanK HIV Hospital General Universitario Gregorio Marañón: M Ángeles Muñoz-Fernández, Irene Consuegra Fernández.

Hospital General Universitario de Alicante (Alicante): Esperanza Merino, Gema García, Irene Portilla, Iván Agea, Joaquín Portilla, José Sánchez-Payá., Juan Carlos Rodríguez, Lina Gimeno, Livia Giner, Marcos Díez, Melissa Carreres, Sergio Reus, Vicente Boix, Diego Torrús.

Hospital Universitario de Canarias (San Cristóbal de la Laguna): Ana López Lirola, Dácil García, Felicitas Díaz-Flores, Juan Luis Gómez, María del Mar Alonso, Ricardo Pelazas., Jehovana Hernández, María Remedios Alemán, María Inmaculada Hernández.

Hospital Universitario Central de Asturias (Oviedo): Víctor Asensi, Eulalia Valle, María Eugenia Rivas Carmenado, Tomás Suárez-Zarracina Secades, Laura Pérez Is.

Hospital Universitario 12 de Octubre (Madrid): Rafael Rubio, Federico Pulido, Otilia Bisbal, Asunción Hernando, Lourdes Domínguez, David Rial Crestelo, Laura Bermejo, Mireia Santacreu.

Servicio de Enfermedades Infecciosas. Hospital Universitario Donostia. Instituto de Investigación BioDonostia. (Donostia-San Sebastián): José Antonio Iribarren, Julio Arrizabalaga, María José Aramburu, Xabier Camino, Francisco Rodríguez-Arrondo, Miguel Ángel von Wichmann, Lidia Pascual Tomé, Miguel Ángel Goenaga, Mª Jesús Bustinduy, Harkaitz Azkune, Maialen Ibarguren, Aitziber Lizardi, Xabier Kortajarena, Mª Pilar Carmona Oyaga, Maitane Umerez Igartua.

Hospital General Universitario De Elche (Elche): Félix Gutiérrez, Mar Masiá, Sergio Padilla, Catalina Robledano, Joan Gregori Colomé, Araceli Adsuar, Rafael Pascual, Marta Fernández, José Alberto García, Xavier Barber, Vanessa Agullo Re, Javier Garcia Abellán, Reyes Pascual Pérez, María Roca**.**

Hospital Universitari Germans Trias i Pujol (Can Ruti) (Badalona): Roberto Muga, Arantza Sanvisens, Daniel Fuster.

Hospital General Universitario Gregorio Marañón (Madrid): Juan Berenguer, Juan Carlos López Bernaldo de Quirós, Isabel Gutiérrez, Margarita Ramírez, Belén Padilla, Paloma Gijón, Teresa Aldamiz-Echevarría, Francisco Tejerina, Francisco José Parras, Pascual Balsalobre, Cristina Diez, Leire Pérez Latorre, Chiara Fanciulli.

Hospital Universitari de Tarragona Joan XXIII (Tarragona): Francesc Vidal, Joaquín Peraire, Consuelo Viladés, Sergio Veloso, Montserrat Vargas, Montserrat Olona, Anna Rull, Verónica Alba, Elena Yeregui, Jenifer Masip, Laia Reverté.

Hospital Universitario y Politécnico de La Fe (Valencia): Marta Montero Alonso, José López Aldeguer, Marino Blanes Juliá, María Tasias Pitarch, Iván Castro Hernández, Eva Calabuig Muñoz, Sandra Cuéllar Tovar, Miguel Salavert Lletí, Juan Fernández Navarro.

Hospital Universitario La Paz/IdiPAZ: Juan González-Garcia, José Ramón Arribas, Carmen Busca, Joanna Cano, Julen Cardiñanos, Juan Miguel Castro, Ana Delgado Hierro, Luis Escosa, Iker Falces Romero, Pedro Herranz, Víctor Hontañón, Milagros García López-Hortelano, Alicia González-Baeza, Rosa de Miguel, Maria Luz Martín-Carbonero, Mario Mayoral, Maria Jose Mellado, Rafael Micán, Rocio Montejano, María Luisa Montes, Victoria Moreno, Ignacio Pérez-Valero, Luis Ramos Ruperto, Berta Rodés, Talia Sainz, Elena Sendagorta, Eulalia Valencia.

Hospital San Pedro Centro de Investigación Biomédica de La Rioja (CIBIR) (Logroño): José Ramón Blanco, José Antonio Oteo, Valvanera Ibarra, Luis Metola, Mercedes Sanz, Laura Pérez-Martínez.

Hospital Universitario Miguel Servet (Zaragoza): Piedad Arazo, Gloria Sampériz.

Hospital Universitari MutuaTerrassa (Terrasa): David Dalmau, Angels Jaén, Montse Sanmartí, Mireia Cairó, Javier Martinez-Lacasa, Pablo Velli, Roser Font, Marina Martinez, Francesco Aiello.

Complejo Hospitalario de Navarra (Pamplona): Maria Rivero Marcotegui, Jesús Repáraz, María Gracia Ruiz de Alda, María Teresa de León Cano, Beatriz Pierola Ruiz de Galarreta.

Corporació Sanitària Parc Taulí (Sabadell): María José Amengual, Gemma Navarro, Manel Cervantes Garcia, Sonia Calzado Isbert, Marta Navarro Vilasaro.

Hospital Universitario de La Princesa (Madrid): Ignacio de los Santos, Jesús Sanz Sanz, Ana Salas Aparicio, Cristina Sarria Cepeda, Lucio Garcia-Fraile Fraile, Enrique Martín Gayo.

Hospital Universitario Ramón y Cajal (Madrid): Santiago Moreno, José Luis Casado Osorio, Fernando Dronda Nuñez, Ana Moreno Zamora, Maria Jesús Pérez Elías, Carolina Gutiérrez, Nadia Madrid, Santos del Campo Terrón, Sergio Serrano Villar, Maria Jesús Vivancos Gallego, Javier Martínez Sanz, Usua Anxa Urroz, Tamara Velasco, Alejandro Vallejo.

Hospital General Universitario Reina Sofía (Murcia): Enrique Bernal, Alfredo Cano Sanchez, Antonia Alcaraz García, Joaquín Bravo Urbieta, Ángeles Muñoz Perez, Maria Jose Alcaraz, Maria del Carmen Villalba.

Hospital Nuevo San Cecilio (Granada): Federico García, José Hernández Quero, Leopoldo Muñoz Medina, Marta Alvarez, Natalia Chueca, David Vinuesa García, Clara Martinez-Montes, Carlos Guerrero Beltrán, Adolfo de Salazar Gonzalez, Ana Fuentes Lopez.

Centro Sanitario Sandoval (Madrid): Jorge Del Romero, Montserrat Raposo Utrilla, Carmen Rodríguez, Teresa Puerta, Juan Carlos Carrió, Mar Vera, Juan Ballesteros, Oskar Ayerdi.

Hospital Clínico Universitario de Santiago (Santiago de Compostela): Antonio Antela, Elena Losada.

Hospital Universitario Son Espases (Palma de Mallorca): Melchor Riera, María Peñaranda, Mª Angels Ribas, Antoni A Campins, Carmen Vidal, Francisco Fanjul, Javier Murillas, Francisco Homar, Helem H Vilchez, Maria Luisa Martin, Antoni Payeras.

Hospital Universitario Virgen de la Victoria (Málaga): Jesús Santos, Cristina Gómez Ayerbe, Isabel Viciana, Rosario Palacios, Carmen Pérez López, Carmen Maria Gonzalez-Domenec.

Hospital Universitario Virgen del Rocío (Sevilla): Pompeyo Viciana, Nuria Espinosa, Luis Fernando López-Cortés**.**

Hospital Universitario de Bellvitge (Hospitalet de Llobregat): Daniel Podzamczer, Arkaitz Imaz, Juan Tiraboschi, Ana Silva, María Saumoy, Paula Prieto.

Hospital Universitario Valle de Hebrón (Barcelona): Esteban Ribera, Adrian Curran.

Hospital Costa del Sol (Marbella): Julián Olalla Sierra, Javier Pérez Stachowski, Alfonso del Arco, Javier de la torre, José Luis Prada, José María García de Lomas Guerrero.

Hospital General Universitario Santa Lucía (Cartagena): Onofre Juan Martínez, Francisco Jesús Vera, Lorena Martínez, Josefina García, Begoña Alcaraz, Amaya Jimeno.

Complejo Hospitalario Universitario a Coruña (Chuac) (A Coruña): Ángeles Castro Iglesias, Berta Pernas Souto, Álvaro Mena de Cea.

Hospital Universitario Basurto (Bilbao): Josefa Muñoz, Miren Zuriñe Zubero, Josu Mirena Baraia-Etxaburu, Sofía Ibarra Ugarte, Oscar Luis Ferrero Beneitez, Josefina López de Munain, Mª Mar Cámara López, Mireia de la Peña, Miriam Lopez, Iñigo Lopez Azkarreta.

Hospital Universitario Virgen de la Arrixaca (El Palmar): Carlos Galera, Helena Albendin, Aurora Pérez, Asunción Iborra, Antonio Moreno, Maria Angustias Merlos, Asunción Vidal, Marisa Meca.

Hospital de la Marina Baixa (La Vila Joiosa): Concha Amador, Francisco Pasquau, Javier Ena, Concha Benito, Vicenta Fenoll, Concepción Gil Anguita, José Tomás Algado Rabasa.

Hospital Universitario Infanta Sofía (San Sebastián de los Reyes): Inés Suárez-García, Eduardo Malmierca, Patricia González-Ruano, Dolores Martín Rodrigo, Mª Pilar Ruiz Seco.

Hospital Universitario de Jaén (Jaén): Mohamed Omar Mohamed-Balghata, María Amparo Gómez Vidal.

Hospital San Agustín (Avilés): Miguel Alberto de Zarraga.

Hospital Clínico San Carlos (Madrid): Vicente Estrada Pérez, Maria Jesús Téllez Molina, Jorge Vergas García, Juncal Pérez-Somarriba Moreno.

Hospital Universitario Fundación Jiménez Díaz (Madrid): Miguel Górgolas, Alfonso Cabello, Beatriz Álvarez, Laura Prieto.

Hospital Universitario Príncipe de Asturias (Alcalá de Henares): José Sanz Moreno, Alberto Arranz Caso, Cristina Hernández Gutiérrez, María Novella Mena.

Hospital Clínico Universitario de Valencia (València): María José Galindo Puerto, Ramón Fernando Vilalta, Ana Ferrer Ribera.

Hospital Reina Sofía (Córdoba): Antonio Rivero Román, Antonio Rivero Juárez, Pedro López López, Isabel Machuca Sánchez, Mario Frias Casas, Angela Camacho Espejo.

Hospital Universitario Severo Ochoa (Leganés): Miguel Cervero Jiménez, Rafael Torres Perea.

Nuestra Señora de Valme (Sevilla): Juan A Pineda, Pilar Rincón Mayo, Juan Macías Sanchez, Nicolás Merchante Gutierrez, Luis Miguel Real, Anais Corma Gomez, Marta Fernández Fuertes, Alejandro Gonzalez-Serna.

Hospital Álvaro Cunqueiro (Vigo): Eva Poveda, Alexandre Pérez, Manuel Crespo, Luis Morano, Celia Miralles, Antonio Ocampo, Guillermo Pousada.
